# Environmental health perceptions of urban youth from low‐income communities: A qualitative photovoice study and framework

**DOI:** 10.1111/hex.13776

**Published:** 2023-06-14

**Authors:** Nadav L. Sprague, Hannah M. Zonnevylle, Lexi Jackson Hall, Rosalind Williams, Hannah Dains, Donghai Liang, Christine C. Ekenga

**Affiliations:** ^1^ Department of Epidemiology, Mailman School of Public Health Columbia University New York City New York USA; ^2^ Gateway to the Great Outdoors St. Louis Missouri USA; ^3^ Gangarosa Department of Environmental Health, Rollins School of Public Health Emory University Atlanta Georgia USA

**Keywords:** environmental exposures, environmental justice, outdoor education, participatory action research

## Abstract

**Background:**

Children are amongst the most susceptible groups to environmental exposures, for both immediate and life‐course health outcomes. Despite their increased susceptibility, children's knowledge, experiences and voices are understudied. A deeper understanding of children's environmental health perceptions has the potential to better inform policy, develop targeted interventions and improve public health outcomes.

**Methods:**

In this study, our community–academic partnership used the Photovoice research method to examine how urban children from low‐income communities perceive environmental influences on their health. Twenty children, ages 10–12, took photographs and participated in focus group interviews regarding their perspectives on how the environment influences their health.

**Results:**

Qualitative analyses revealed five major thematic categories: environmental exposures, environmental health sentiments, environmental health outcomes, interest in environmental health and environmental health solutions. We used the findings to develop an environmental health perspective theoretical framework that can inform future work designed to promote the environmental health and well‐being of children from low‐income communities in urban communities.

**Conclusion:**

Photovoice enabled children from low‐income communities to capture and communicate their environmental health perceptions. These findings have the potential to inform and identify potential targets and opportunities for environmental health interventions and promotion in their communities.

**Patient or Public Contribution:**

Partnerships with community‐based organizations were central to the present study. By design, these community‐based partners were involved in the conduct and procedures of the study.

## 1 INTRODUCTION

Children are amongst the most susceptible groups to environmental exposures.^1^ Childhood environmental exposures have significant implications for immediate health outcomes as well as health outcomes throughout the life course.^2^ In the United States (US), there are striking racial, ethnic, and socioeconomic disparities in childhood environmental exposures.^3^ Specifically, compared to their White, non‐Hispanic, or high‐income counterparts, Black, Hispanic, or children from low‐income communities consistently experience higher exposure to environmental hazards and lower exposures to health‐promotive environmental facets.^4^, ^5^ Such environmental hazards include exposure to lead‐based paint, tobacco smoke, ambient and traffic‐related air pollution, and living in closer proximity to hazardous waste.^6^ Examples of promotive environmental facets include nature contact, tree shade, clean neighbourhoods, and access to healthy food.^7^, ^8^ Furthermore, individual and community‐level perceptions of the environment and associated health impacts play an important role in how individuals engage with their surroundings.^9^, ^10^, ^11^ Environmental health perceptions may influence the diet, physical activity, and physical safety of community members, especially children, and thus impact their development and behaviours later in life.^6^, ^12^ Currently, there is a substantial knowledge gap in the literature as children's environmental health perceptions are critically understudied. Researchers must engage, examine, and incorporate children's environmental health perceptions into public health and public policy discourse to better improve health outcomes, especially in urban and low‐income communities.

Photovoice is a community‐based participatory action research methodology in which researchers can understand and incorporate childhood environmental health perceptions.^13^, ^14^, ^15^ Briefly, Photovoice engages research participants by asking them to take photos on a given topic and later asking them to discuss these photos during a focus group.^13^, ^14^, ^15^ The Photovoice research method has been traditionally viewed as an avenue through which marginalized communities can participate in academic research and ultimately have influence over policy decisions.^13^, ^16^, ^17^ Photovoice allows for collaboration between community members and researchers by empowering community members to capture and communicate their perceptions and knowledge.^18^

Photovoice has been shown to be an effective tool for empowering children and youth.^15^, ^19^, ^20^ For example, Photovoice enabled minority New York City youth to reflect on food justice issues and engage in promoting positive community changes.^16^ Photovoice has also been used as a participatory process for research and social change allowing children to feel ‘seen’ by adults.^19^ Given its participatory focus, Photovoice may be a valuable tool for allowing children to express their environmental health concerns and perceptions. Therefore, the purpose of the current study was to identify and characterize the environmental health perceptions of urban, low‐income US children. We utilized Photovoice methods to engage a sample of children from St. Louis, Missouri, to identify and better understand their perspectives on environmental exposures and health concerns. We used the results to develop a youth‐informed environmental health perspective theoretical framework.

## 2 METHODS

### 2.1 Study context

A community‐academic partnership between Gateway to the Great Outdoors, Columbia University's Mailman School of Public Health, Emory University Rollins School of Public Health and Washington University in St. Louis was established in 2017 to provide environmental education programming to St. Louis Public Schools, which has a student body that is 95% or more on the free and reduced lunch programme.^15^, ^21^, ^22^ This partnership was established as St. Louis Public School students face disproportionate amounts of environmental and behavioural health disparities compared to students in neighbouring school districts.^23^ The environmental education intervention run by this community–academic partnership has been described in detail in prior publications.^15^, ^21^ Briefly, the intervention consists of (1) weekly interactive, in‐class science, technology, engineering, art and math lessons focused on environmental health and (2) monthly nature‐based field trips.

The current study was approved by the Human Subjects Review Boards at Emory University, Columbia University, and Washington University in St. Louis, and informed consent or assent was obtained from all study participants. The in‐person intervention was administered during the Fall 2020 semester (August–December, 10 weekly lessons and 3 nature‐based field trips) and during the Spring 2021 semester (January–May, 10 weekly lessons and 3 nature‐based field trips), to children attending the same St. Louis Public School District school. All students participating in the community‐academic partnership's in‐person programming during the 2020–2021 academic school year were included in this study.

The research team was multidisciplinary, consisting of researchers trained in epidemiology, biology, anthropology, environmental science and environmental education. The multidisciplinary research team included a senior author (C. C. E.) with lived experience as a Black woman growing up in St. Louis. She has an interest in issues of environmental justice for Black US communities. The senior author provided supervision and critical feedback throughout the research process. Data collection and analyses were completed by N. L. S., H. M. Z., L. J. H., R. W. and H. D. who do have a shared history with study participants. All authors aimed to amplify the voices of the child study participants, whose perspectives are often underexamined in environmental health promotion research.

### 2.2 Photovoice environmental education intervention

The students participated in a Photovoice community‐based participatory research project (Photovoice activity). During the first class of the intervention, the students received disposable cameras and then participated in a 60‐min lesson on how to use said cameras as well as how to obtain consent when photographing other humans. After the 60‐min lesson, the students were instructed to take photographs of how the environment impacts their health. The students returned their cameras on week 2 of the intervention. On week 3, the developed photographs were brought to the classroom and the students were individually interviewed about the photographs that they took by trained research assistants.

We conducted individual interviews instead of focus groups because children may have been less likely to disclose their perspectives with their peers present. The interviews were semistructured interviews to explore children's perspectives in depth. Sixty minutes were allotted for the interviews during week 3 of the intervention. Interview questions focused on what the student captured in the photograph, how the photograph related to environmental health and how the photograph made the child feel (an interview guide is available upon request from the senior author). The interviews, which ranged from 20 to 52 min long, were recorded and later transcribed.

### 2.3 Data analysis

The study used a pragmatism paradigm.^24^ As such, we undertook a mixed thematic analysis approach that aimed to identify and characterize environmental health perceptions through both codebook and coding reliability approaches. Our thematic analysis approach was adapted from Castleberry and Nolen^25^ and Braun and Clarke.^26^ Briefly, Castleberry and Nolen's approach, widely used in the health sciences, consists of five steps to thematic analysis: compiling, disassembling, reassembling, interpreting and concluding.^25^ Our approach also utilized what Braun and Clarke term ‘coding reliability’ and ‘codebook’ thematic analysis.^26^ These approaches allowed for both a detailed accounting of participants' environmental health perceptions (coding reliability) and inductive theme development (codebook).

Four trained researchers familiarized themselves with the data through a preliminary analysis of the transcripts. Then, the four researchers independently brainstormed potential thematic codes based on the transcripts. After, the four researchers met and discussed their potential thematic codes. From that meeting, the researchers developed an official list of themes that were derived from the manuscript. A fifth researcher, who was purposefully excluded from the initial meetings, revised and approved the final list of themes. Once the final list of themes was approved, two researchers independently coded every transcript for every theme. Agreement between the two independent researcher's coding was determined through the *κ* statistic. The *κ* statistic ranges from −1.0 (complete disagreement) to 1.0 (complete agreement); where scores of 0.61–0.80 suggest substantial agreement and scores of 0.81–1.0 suggest strong agreement.^27^, ^28^ All transcripts were coded and analyzed in Microsoft Word and Microsoft Excel. We utilized the guidelines outlined in the Standards for Reporting of Qualitative Research checklist in reporting this study (Supporting Information: Appendix 1).^29^

Lastly, using the subjectivist inductive approach to research, results from this study were used to develop a youth‐informed environmental health perspectives theoretical framework.^30^ The framework is presented in the discussion section of this paper.

## 3 RESULTS

A total of 20 students participated in the current study. Of the 20 participants, 8 (40.0%) students were Black, 5 (25.0%) were White, 1 (5.0%) was Asian, 4 (20%) were two or more races and 2 (10.0%) were self‐identified as other. The median age of the study participants was 12.

The thematic categories from the qualitative data analysis are presented in Table 1. There were five major thematic categories: environmental exposures, environmental health sentiments, environmental health outcomes, interest in environmental health and environmental health solutions. The interrater agreement for each subtheme ranged from a *κ* of 0.72 for environmental exposures and 0.93 for interest in environmental health.

**Table 1:** Themes and subthemes from Photovoice focus groups.

Theme	Subtheme
Environmental exposure	Nature and greenspace
	Built environment
	Climate change
	Pollution and waste management
	Food environment
	Violence
Environmental health perceptions	Positive
	Negative
Environmental health outcomes	Emotional and mental health
	Physical activity
	Safety
Interest in environmental health	Inquisitive
	Apathetic
Environmental health solutions	Individual level
	Community level
	Global Level

### 3.1 Environmental exposures

**Figure 1:**
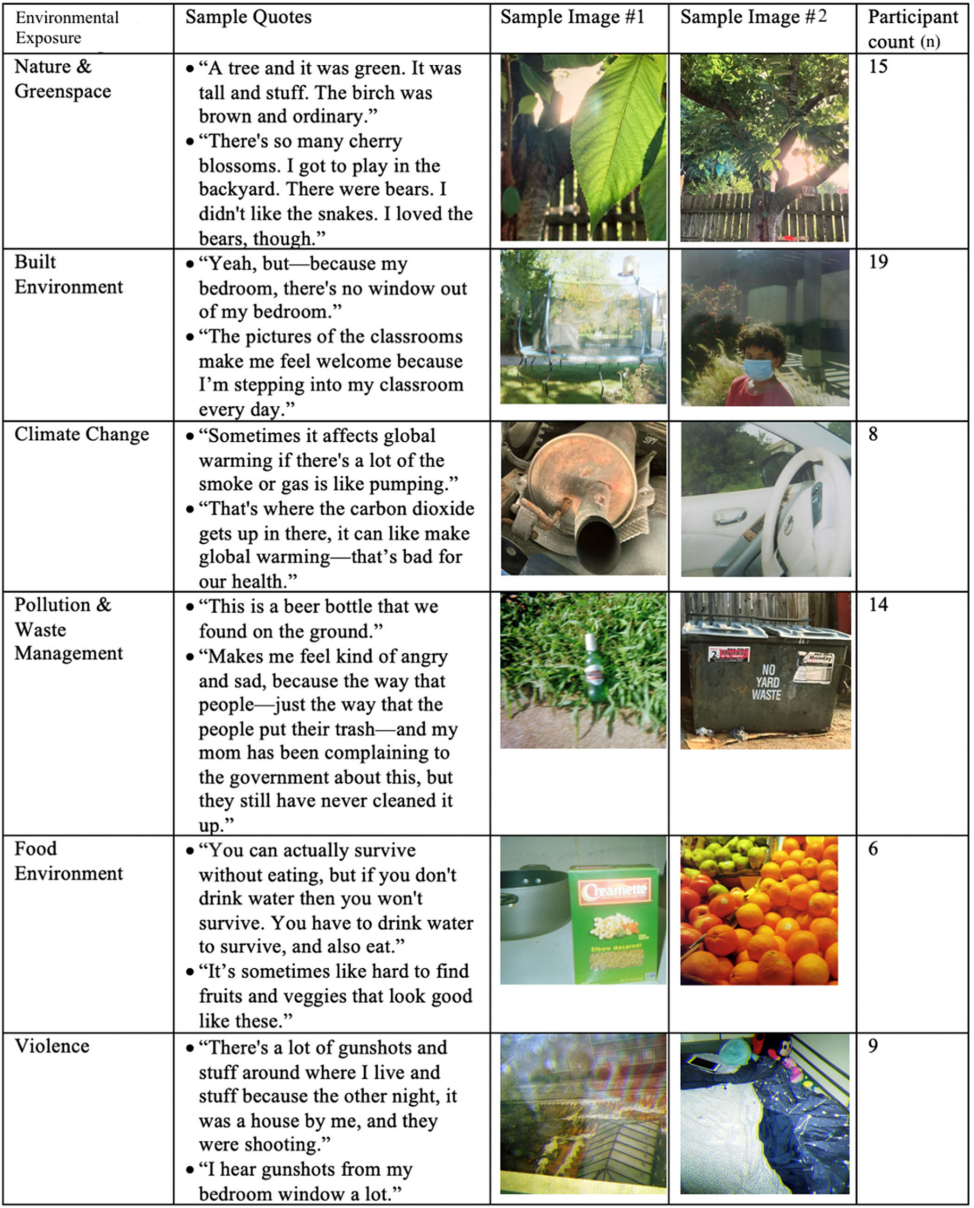
Sample quotes and images of the environmental exposure subtheme.

The environmental exposures theme was characterized by photographs of the students' environment which they believed influenced their health. This theme included the following subthemes: nature and greenspace, built environment, climate change, pollution and waste management, food environment and violence. The environmental exposure subthemes, sample quotes and sample images are presented in Figure 1.

#### 3.1.1 Nature and greenspace

Encounters with nature and greenspace, such as parks and trees in their neighbourhoods, were described by study participants. One student stated that they ‘took pictures of nature. I took pictures of leaves. I took pictures of trees, like trees in the cold time. They didn't have no leaves on there. I took pictures of the sky’ (participant 1). This quote demonstrates students' awareness of the nature surrounding them, as well the conditions and appearances of that nature. Another student took a picture of a tree ‘Because a tree can help you calm down and lose stress’ (participant 2). This quote reveals students' understanding of nature and greenspace's impact on their mental health.

#### 3.1.2 Built environment

Participants also described aspects of their environment that were structural or manmade, as opposed to exposures to nature. Examples included participants' schools, homes and other aspects of their built urban environment. One student described enjoying travelling on the highway, saying, ‘I feel kind of happy, 'cause it's sunny, and whenever we're driving to, like, Walmart you can just smell the fresh air as we're driving on the highway, and it just always gives me a good vibe for some reason. I just feel happy knowing we're on the highway’ (participant 3). This quote demonstrates the feelings of joy the student receives when engaging with features of the built environment (a highway and car).

#### 3.1.3 Climate change

Climate change and its influence on health were also discussed. Some students discussed their personal experiences with climate change in their own environment, while others discussed climate change on a global level. For example, one student voiced concerns about the health impacts of forest fires, as she stated ‘Australia is on fire! People are losing their homes and it can't be good for breathing’ (participant 4). This quote shows that students are aware of the global effects of climate change on human health and wellbeing.

#### 3.1.4 Pollution and waste management

Pollution and waste management were identified as environmental exposures that impact health. Specifically, students focused on exposure to air pollution, litter and noise pollution. Students discussed being exposed to air pollution. For example, one participant took a photo of a car's exhaust pipe (participant 5). Another student took a picture of his local Walmart and described the air as ‘polluted’ (participant 3). This reveals that students associate human activities, such as large‐scale production and car exhaust, with environmental and health harm.

Students also took photographs and discussed the accumulation of litter and trash in their environment. One student stated that they would ‘pick all the trash up’ to improve their environment (participant 6). This quote shows that students identify litter as a relevant environmental exposure. Another student described the negative effects of litter, saying ‘Some people are throwing trash on the ground, like this right here, that's going to affect our environment sooner or later. It might take some time, but if this keeps up, it will affect our environment’ (participant 7). This quote demonstrates this student's knowledge of the effects of environmental exposure accumulation. One student said of a picture of a pile of litter, ‘Why this picture is important to me is that you should use a dumpster and not just put trash in a pile. Just use it like a pile like—just the ground as a dumpster. You should use it like a trash can’ (participant 8). Another participant (4), who took a photo of a recycling bin, said ‘Well, the recycling that—'cause reuse—the three Rs, we can help reduce, reuse, and recycle, of course, and that's good for the environment and it helps the environment’. The two previous quotes show that students are knowledgeable about the proper management of trash as an environmental exposure. Participants' descriptions of exposure to noise pollution were also categorized under this subtheme. One participant referenced gunshots as a source of noise pollution.

#### 3.1.5 Food environment

Participants' mentions of nutrition and diet, and the effects of nutrition on their health, were categorized in the nutrition subtheme. One student took a picture of water and stated that ‘water is actually really healthy for you, and also you can actually survive without eating, but if you don't drink water then you won't survive. You have to drink water to survive, and also eat’ (participant 9). In this quote, the student is drawing connections between nutritional exposures and health outcomes.

#### 3.1.6 Violence

Participants' references to violence in their environment were categorized under this subtheme. One student referenced gunshots in a nearby house, stating ‘I feel bad about it because there's a lot of gunshots and stuff around where I live and stuff’ (participant 10). This quote reveals that students view violence in their community as an environmental exposure that directly impacts their mental health.

### 3.2 Environmental health sentiments

This thematic category was characterized by the direction in which participants viewed how their environment influenced their health outcomes. We coded these emotions into two subthemes: positive and negative.

#### 3.2.1 Positive sentiments

When describing pictures of their environment, many participants expressed positive emotions toward the people, places and natural objects captured in their photos. Several participants shared pictures of their friends and family, while others captured outdoor spaces in their community. When asked how her picture of a tree made her feel, one participant answered, ‘It just makes me feel [a] sense of worth’, (participant 11) indicating that interaction with nature can improve feelings of self‐efficacy and personal worth. Several participants credited their environments for a positive impact on their mental, physical, or emotional health. One participant described her feelings towards her picture of spinach as ‘excited’ because ‘it helps me with my health, and also spinach is actually really good’ (participant 12). This quote highlights the positive emotions that students feel when they enjoy activities that they also identify as health promotive.

#### 3.2.2 Negative sentiments

Several participants expressed negative emotions toward their environmental health when describing their photos. Much of the negative sentiment stemmed from the presence of environmental harm or the absence of environmental amenities. One participant lamented that ‘environments with not enough grass bother me for lots. The smells of the city are horrible and I hate it’ (participant 2). The quote reveals environmental exposures within their city that students feel negatively towards. Others expressed negative sentiments towards their environment due to safety issues like violence and shootings. Another common cause of negative sentiment was the presence of litter in their environments. When asked why it was important to him to capture a picture of litter, one participant responded, ‘the trash one is important to me because it affects the earth. It affects me because I don't like seeing trash out. It just looks messy’ (participant 7). This quote demonstrates that both health and aesthetic conditions influence students' feelings toward their environment.

### 3.3 Environmental health outcomes

Participants' perceptions of the health effects from environmental exposures were categorized under the environmental health outcomes theme. This theme included three subthemes: emotional and mental health, physical activity and safety.

#### 3.3.1 Emotional and mental health

Many students expressed that their environment affected their emotional and mental health, both positively and negatively. Many students believed that being in nature had positive impacts on their mental health. For example, one student stated that nature enhances his mental health ‘cause the sun is always shining and stuff, so yeah, it affects me in a good way’ (participant 13). This quote reveals that students acknowledge that their environmental exposures may positively impact their mental health outcomes. Another student stated that a tree in her backyard ‘makes me feel special’ (participant 2). This is another comment on how greenspace may promote positive mental health benefits for students by increasing their sense of worth. Conversely, pollution and litter in the students' environment negatively influenced their emotional and mental health. For example, one student took a photograph of trash in his neighbourhood and said that ‘It makes me feel kind of angry and sad, because the way that people—just the way that the people put their trash… It just makes me angry and sad. It makes me sad that people just put trash there for no reason’ (participant 6). This quote shows that the presence of trash as well as human behaviour towards the environment can negatively impact students' mental health.

#### 3.3.2 Physical activity

Students also expressed that their environment influenced their physical activity level. For example, one student describes exercising in their home environment alone in their bedroom: ‘I don't tell my mom these things, but I actually practice my jumping, kicking, punching, and chopping on my doors and rug, and practising my wall walk’ (participant 14). In this quote, the student reveals environmental features (doors, rugs, privacy) that promote physical activity. Another student took a photo of a trampoline in their yard, ‘This is a trampoline. I say it helps your health because if you jump on it, you can get exercise and that gets your heart pumpin' so it can help your health’ (participant 15). In this quote, the student identifies a feature of their built environment (a trampoline) that directly impacts their physical activity levels.

#### 3.3.3 Safety

Some students discussed how their environment influenced their safety. Some students described feeling safe in their environments, while others described feeling unsafe. One student described being happy about having a place to live, saying ‘'Cause if we didn't have something to live, we might be in danger’ (participant 8), This reveals that students directly tie their environment to safety. Another student described gunshots as part of their environment: ‘the other night, it was a house by me and they were shooting. I don't know why’ (participant 16). This quote shows that students may have safety concerns about their surrounding environments.

### 3.4 Interest in environmental health topics

Participants expressed differing levels of engagement and curiosity toward their environment and its effect on their health. We coded these emotions into two subthemes: inquisitive and apathetic.

#### 3.4.1 Inquisitive

Participants expressed curiosity toward the elements captured in their photos and how they might affect their health. When asked how the trees she captured affected her health, one participant (1) asked, ‘Don't trees give you oxygen?’ This quote shows students asking inquisitive questions to clarify how their environmental surrounding impacts their health. Other participants made observations and/or hypothesized about their environmental health based on their photos. One participant conjectured that the build‐up of trash she observed in her environment would ‘affect our environment sooner or later’ (participant 7). This quote highlights that students can draw conclusions based on prior knowledge and forecast future outcomes.

#### 3.4.2 Apathetic

Participants also expressed apathy towards their photos and/or the concept of environmental health in general. When asked why their photos were important to them, some participants could not name a reason. Others declared their photos unimportant altogether. When asked how the environment affects their health, seven participants responded with ‘I don't know’. This response indicates that some participants had never previously considered the impact of their environment on their health and did not express interest in doing so during the interview. While the participants had feelings of apathy toward how their environment impacts their health, they were able to discuss their photographs in detail.

### 3.5 Environmental health solutions

Several participants (*n* = 17) offered solutions to improve their environments and promote positive health outcomes. These solutions ranged in scale and mentioned a variety of stakeholders. We coded these solutions into three subthemes: individual level, community level and global.

#### 3.5.1 Individual‐level solutions

Some participants offered examples of actions they could take as individuals to improve their environments and promote positive health outcomes. Most examples focused on the elimination of trash and litter. When asked how they protect their environment, one participant answered, ‘I try to pick up trash when I can. Every time my mom or my granny wants to litter without noticing it, I tell them, “No, don't litter”’ (participant 18). In this quote, the student is highlighting their ability to take individual‐level action to prevent littering and reduce the negative environmental exposure of litter. Several participants expressed the desire for more environmental amenities to improve their health outcomes. When asked if they would change anything about his environment, another participant noted, ‘I would plant more trees’ (participant 19). This quote highlights a specific action that a student would do to promote health in their environment.

#### 3.5.2 Community‐level solutions

Other participants offered ideas to improve their community, neighbourhood or city. These solutions would often require a group actor, such as a government or neighbourhood association, to offer more environmental amenities. For example, one participant stated that they would, ‘…get more trees and have all the houses more away from each other. I don't know. Just have more trees, more grass, and just more stuff where people can breathe better and just have more space to have fun and stuff, I guess, outside’ (participant 1). In this quote, the student is demonstrating a community‐level intervention to improve overall environmental health.

#### 3.5.3 Global solutions

Some participants offered solutions to improve the environment on a global scale. One participant showed a picture of the tailpipe on their family's car and said, ‘…if we continue to change from this to electric cars, all‐electric cars, then we can stop all the pollution’ (participant 20). They predicted that electric cars would become the standard around the globe within the next few decades.

## 4 DISCUSSION

**Figure 2:**
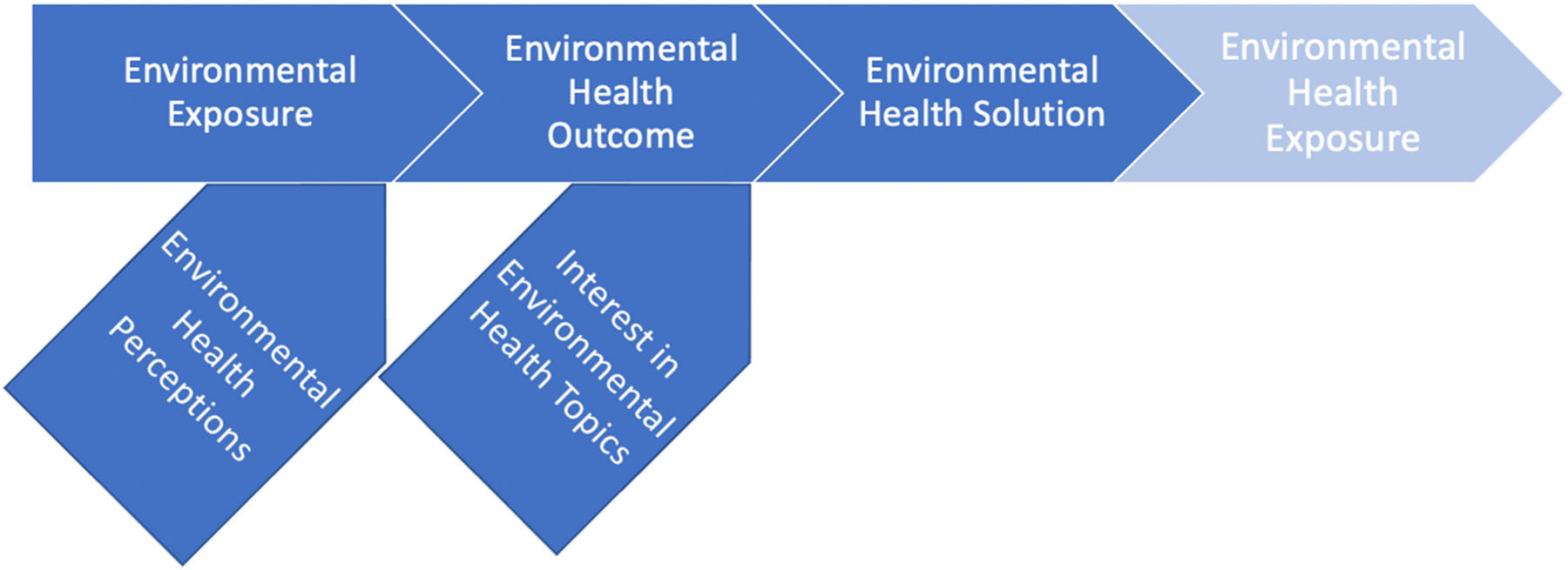
Youth‐informed environmental health theoretical framework.

This study examined the environmental health perceptions of urban children from low‐income communities through the Photovoice research methods. We chose to use the Photovoice research method as this and other community engagement research strategies are paramount for communicating associated health risks with those living in areas of poor environmental health.^31^ The qualitative analysis of the Photovoice method revealed five major thematic categories: (1) environmental exposures, (2) environmental health sentiments, (3) environmental health outcomes, (4) interest in environmental health and (5) environmental health solutions. These findings have informed the development of a theoretical framework to characterize the environmental health perspectives of children from low‐income households in urban settings (Figure 2).

We believe that this study is one of the first efforts to incorporate the knowledge and perspectives of children from low‐income US communities to inform the development of an environmental health perspective theoretical framework. Although community‐engaged studies often incorporate the experiences of community members, very few focus on the perspectives of children. Prior work has shown that environmental perceptions have just as large of an impact on health outcomes as actual environmental exposures.^32^, ^33^ This study innovatively incorporates the knowledge and perceptions of youth to develop an environmental health perspective theoretical framework. Our child‐informed environmental health perspective theoretical framework will inform future research and intervention efforts with children from low‐income households in St. Louis.

As depicted in Figure 2, both environmental exposures and environmental health sentiments influence environmental health outcomes. Existing research suggests that environmental exposures may promote both positive and negative health outcomes.^34^, ^35^ For example, studies have found that nature and greenspace exposures improve a wide array of health outcomes (such as sleep, mental health, well‐being, attention, cardiovascular, pain, vision and respiratory).^36^, ^37^ Alternatively, exposure to environmental toxins may cause negative health outcomes.^38^ For example, living in a neighbourhood with limited access to supermarkets and fresh produce has been associated with increased risks for obesity and related health outcomes.^39^, ^40^ Exposures to ambient and household air pollution have been associated with a number of adverse health outcomes (including asthma, respiratory disease, cardiovascular disease, lung cancer and mortality).^41^, ^42^ There is a strong existing body of evidence base supporting the causal association between environmental exposures and health outcomes.

Research has also highlighted the significant impact of environmental health sentiments on environmental health outcomes.^32^, ^33^ One study investigated the relationship between environmental exposures, perceptions, and outcomes for indoor and outdoor air quality in Seoul, Korea by collecting data and surveying 396 elementary school students (average age = 11) and 64 parents.^32^ This study found that environmental health sentiments significantly influenced environmental health outcomes, even in areas with comparable environmental exposures.^32^ The current evidence base supports our child‐inform theoretical framework, with both environmental exposures and perceptions influencing outcomes (Figure 2).

Also shown in the theoretical framework (Figure 2), environmental health outcomes ideally lead to multilevel (individual, community, global) environmental health solutions. However, the creation of environmental health solutions is dependent on the level of interest in environmental health issues (inquisitive or apathetic) amongst individuals, activists, educators, communities and/or legislators. For example, for several decades, the negative effects of lead exposure on children's health (such as cognition, school performance and mortality) have been well established.^43^ Despite the knowledge of the negative impacts of lead poisoning, the United States has continued to see extensive human exposure to lead poisoning, threatening the health and livelihood of children.^44^ While the effects of lead on human health were well established, interest in children's environmental health issues increased during the 2014–2015 crisis of lead‐contaminated drinking water in Flint Michigan.^44^ This crisis elevated the awareness and issue of lead poisoning and ignited a public outcry worldwide.^45^, ^46^ Studies suggest that these events caused an increase in the public interest.^46^ In the United States, this elevated awareness (concern over lead water in drinking water) forced school districts to take action and create environmental health solutions (testing school drinking water and shutting down any drinking fountain with potential lead pollution),^46^, ^47^ leading to calls for more community‐oriented, creative and intersectional solutions. For children, evidence suggests that engaging in environmental health education is an effective intervention for increasing inquisitive interest in young people,^48^ and could thus lead to informed solutions.

Our theoretical framework indicates that environmental health solutions may lead to new, positive environmental exposures (Figure 2). For example, the development of new greenspaces may promote improved mental and physical health outcomes.^49^ One study investigated the changes in children's physical activity patterns before and after a large‐scale playground greening intervention at a low‐income Los Angeles Public School.^49^ The study found a 10.0% decrease in sedentary activity and a 48% increase in vigorous activity participation after the playground greening intervention was complete.^49^ This is an example of an environmental health solution (greening playgrounds) altering the environmental exposure (playgrounds and greenspace) and, in turn, altering environmental health outcomes (physical activity).^49^

The main strengths of this study include its use of participatory research methods to examine children's perceptions and develop a children‐informed environmental health perspective theoretical framework. Using a mixed thematic analysis approach to develop thematic codes allowed for the children's voices to lead the research, rather than deferring solely to a potentially biased predetermined framework. The limitations of this study should also be noted. First, this study was conducted during the COVID‐19 pandemic, which may have limited children's outdoor activities and therefore influenced perceived environmental exposures and sentiments. Another limitation includes the transferability of these study findings. The study was conducted in a sample of children from low‐income families in St. Louis and the theoretical framework may or may not be applicable to children in other settings. Future studies in other study settings with children of different socioeconomic, racial and ethnic backgrounds are warranted.

## 5 CONCLUSION

The knowledge, experiences and voices of children are underexamined in environmental health research. The findings of this study have implications for our understanding of children's perceptions and can assist in informing the work of researchers, educators and health and social professionals who interact with children from low‐income households in St. Louis. We developed an environmental health perspective theoretical framework that can inform future survey instruments that include questions about environmental health exposures, sentiments and/or outcomes. Further, the use of Photovoice and the development of this theoretical framework should be considered as the first steps in a series of research strategies aimed at improving our understanding of child environmental health outcomes through improved measurements and assessments. As such, our findings have the potential to inform and identify potential targets and opportunities for interventions aimed to promote child health in an urban, low‐income community.

## AUTHOR CONTRIBUTIONS

The authors confirm their contribution to the paper as follows: *Study conception and design: Conceptualization*: Nadav L. Sprague and Christine C. Ekenga. *Data curation*: Nadav L. Sprague and Christine C. Ekenga. *Formal analysis*: Nadav L. Sprague, Hannah M. Zonnevylle, Lexi Jackson Hall, Rosalind Williams, Hannah Dains and Christine C. Ekenga. *Funding acquisition*: Nadav L. Sprague and Christine C. Ekenga. *Investigation*: Nadav L. Sprague and Christine C. Ekenga. *Methodology*: Nadav L. Sprague, Hannah M. Zonnevylle and Christine C. Ekenga. *Project administration*: Nadav L. Sprague, Hannah M. Zonnevylle, and Christine C. Ekenga. *Resources*: Nadav L. Sprague and Christine C. Ekenga. *Software*: Nadav L. Sprague and Christine C. Ekenga. *Supervision*: Christine C. Ekenga. *Validation*: Nadav L. Sprague, Hannah M. Zonnevylle and Christine C. Ekenga. *Writing—original draft*: Nadav L. Sprague, Hannah M. Zonnevylle, Lexi Jackson Hall, Rosalind Williams, Hannah Dains, Donghai Liang and Christine C. Ekenga. *Writing—review and editing*: Nadav L. Sprague, Hannah M. Zonnevylle, Donghai Liang and Christine C. Ekenga. All authors have read and agreed to the published version of the manuscript.

## CONFLICT OF INTEREST STATEMENT

The authors declare no conflict of interest.

## ETHICS STATEMENT

This study was approved as exempt from written consent by the Institutional Review Boards at Emory University, Washington University in St. Louis, and Columbia University.

## Supporting information

Supporting information.

## Data Availability

The data generated during the study are available from the corresponding author upon reasonable request.
